# Zein Nanoparticles and Strategies to Improve Colloidal Stability: A Mini-Review

**DOI:** 10.3389/fchem.2018.00006

**Published:** 2018-01-25

**Authors:** Mônica Pascoli, Renata de Lima, Leonardo F. Fraceto

**Affiliations:** ^1^Environmental Chemistry, Institute of Science and Technology, São Paulo State University (UNESP), Sorocaba, Brazil; ^2^Department of Biotechnology, University of Sorocaba, Sorocaba, Brazil

**Keywords:** colloidal stability, thermal treatment, ionic strength, zein, nanoparticles

## Abstract

Zein, a protein extracted from maize, can be employed to easily produce nanoscale particles suitable for use as carrier systems. This review investigates the main methods for obtaining zein nanoparticles, as well as the problems and options available in the development of stable colloidal suspensions. Considerable gaps were identified in the literature concerning this topic, with studies being unclear about the factors that affect the stability of zein particles. In the vast majority of cases, no data are presented in relation to the stability of the formulations over time. It could be concluded that in order to produce a high quality system, detailed evaluation is required, considering factors including the zein concentration, pH, ionic strength, thermal treatment of the protein prior to preparation of the nanoparticles, strategies employing other materials as coatings, and the storage conditions. It is extremely important that these aspects should be considered during product development, prior to commercial-scale manufacture.

## Introduction

Zein is the main protein present in maize, accounting for around 50% of the total protein content. It belongs to the prolamin class and is composed of lipophilic amino acid residues. The α-zein form accounts for over 70% of the total zein protein and is the type that is commercially available (Paliwal and Palakurthi, 2014). It is not used for direct human consumption, due to its negative nitrogen balance and low solubility in water. However, it can be easily converted to spherical colloidal nanoparticles (Patel et al., 2010). Due to its high coating capacity, biodegradability, and biocompatibility, zein has been used in modified release systems for the delivery of enzymes, drugs, and essential oils, among other substances (Lee et al., 2013; da Rosa et al., 2015; Park et al., 2015; Wang et al., 2017).

The purpose of the present work is to provide an overview of the main methods of preparation of zein nanoparticles, as well as the main problems related to the temporal stability of these systems. Possible options for increasing the colloidal stability of zein nanoparticles are presented, together with future perspectives for the development of these carrier systems.

### Preparation of zein nanoparticles

There are many methodologies described in the literature for the preparation of zein nanoparticles used for loading with different active compounds (Supplementary Table 1), including nanoprecipitation, liquid-liquid dispersion, phase separation, and electrospraying. Encapsulation techniques are attractive methods based on precipitation processes (Tarhini et al., 2017).

#### Antisolvent nanoprecipitation, liquid-liquid dispersion, and phase separation techniques

**Figure 1:**
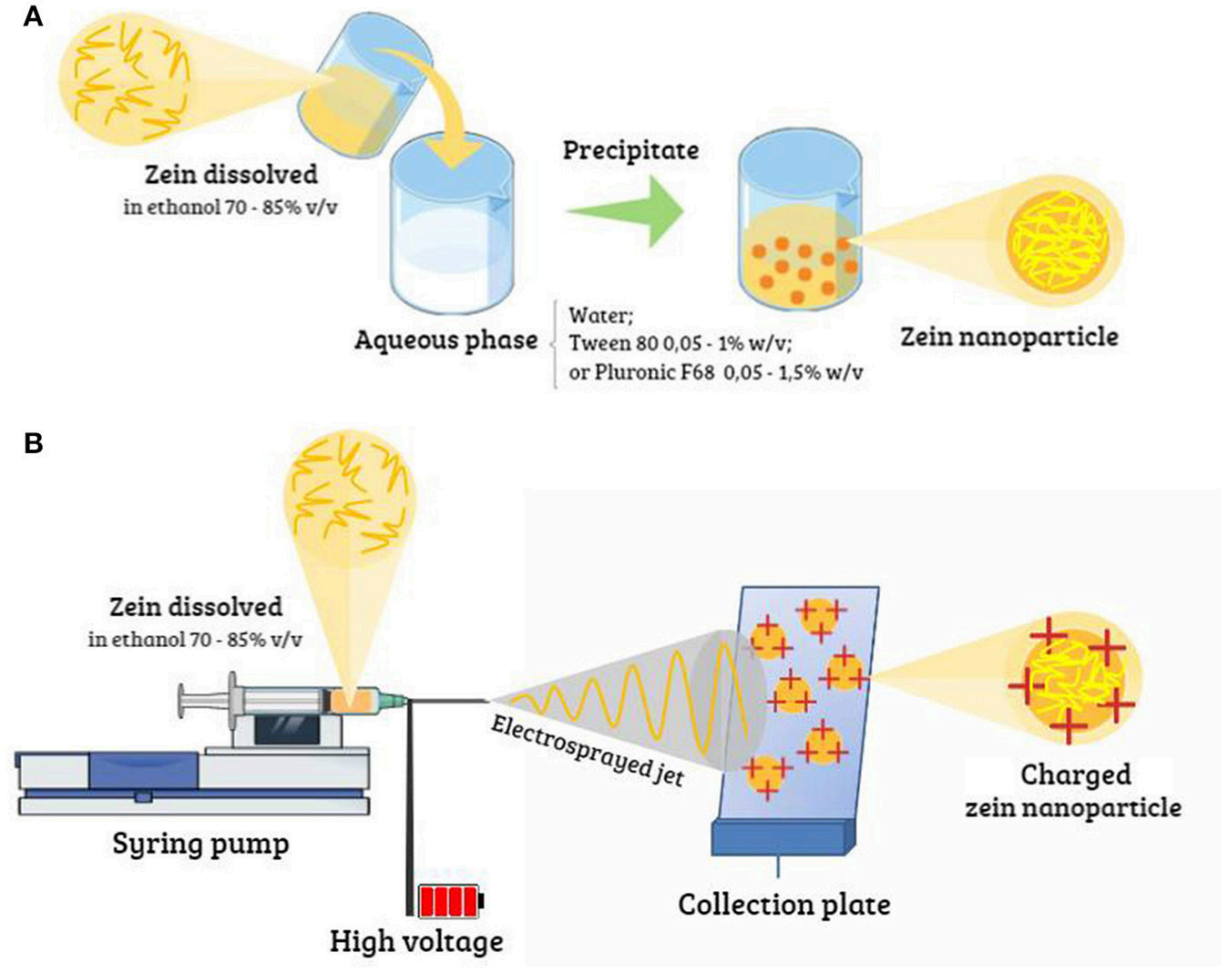
Different methodologies for preparation of zein nanoparticles: **(A)** antisolvent precipitation/liquid-liquid dispersion/phase separation techniques, and **(B)** electrohydrodynamic atomization.

The antisolvent nanoprecipitation technique for the synthesis of nanoparticles has been widely described in the literature (Figure 1A). It is based on the differences in solubility of a protein in different solvents, as a function of pH, ionic strength, and electrolytes. The method involves the addition of a non-solvent to a solution in order to induce supersaturation, leading to precipitation of the solute and the formation of nanoparticles. It is important to select a suitable solvent and antisolvent, considering their miscibility in the concentration range in which they will be used (Li et al., 2013). In this methodology, the nanoparticles formed are dependent on the method and rate of injection of the organic phase into the aqueous phase, the agitation speed, and the volume ratio. There is no need for an emulsifier for particle formation, although its nature and concentration can influence the nanoparticle size (Rao and Geckeler, 2011).

Liquid-liquid dispersion methods consist of the same antisolvent nanoprecipitation method, with the different solubilities of zein in ethanol and water being exploited in order to produce nanoparticles. The interaction of the alcohol and water acts to decrease the concentration of ethanol, hence reducing the solubility of the zein and causing its production in the form of nanoparticles (Zou et al., 2012).

The method described in the literature as phase separation consists of the same procedures described for antisolvent precipitation and liquid-liquid dispersion, followed by centrifugation, separation, and purification of the nanoparticles (Lee et al., 2013).

These different zein nanoparticle production methods involve evaporation of the solvent, which can be achieved by magnetic stirring at room temperature, rotary evaporation, or placing under a flow of nitrogen (Hu and McClements, 2015; Chuacharoen and Sabliov, 2016).

Advantages of these methodologies are that they do not require complex equipment and involve straightforward preparation conditions. They are low cost and provide satisfactory encapsulation efficiencies for active ingredients. However, while these techniques are useful for optimization of formulations using small volumes of samples, the same results may not necessarily be obtained at larger scales.

#### Electrohydrodynamic atomization method

The electrohydrodynamic atomization method (Figure 1B), also known as electrospraying, is based on the separation of a liquid into charged droplets under the influence of an electric field. The liquid passes through a fine metal tube, such as a capillary or a needle, and the liquid meniscus at the tip of the tube is electrically stressed. Nanoparticles with different characteristics can be obtained by varying the electric field strength, the properties of the liquid, and the injection rate. Multiple solutions can be used, with injection of one solution into another, which has the important advantage of producing monodispersed nanoparticles with high encapsulation efficiency (Gomez-Estaca et al., 2012). This method offers faster production of nanoparticles in a single step, making scale-up feasible. However, a disadvantage is the high cost of the production process.

## Colloidal stability of zein nanoparticles

Despite the effectiveness of the methods used to prepare zein nanoparticles, considerable challenges remain concerning the temporal chemical stability of these systems under different storage conditions (Li et al., 2013; Park et al., 2015). Chen and Zhong (2014) studied dispersions of zein nanoparticles and concluded that they presented poor colloidal stability, readily forming aggregates and precipitates in the formulations, hence losing their functionality.

At higher pH, formulations of zein nanoparticles have been found to exhibit aggregation and precipitation (Cheng and Jones, 2017), due to the fact that in solutions with pH above 5, zein is close to its isoelectric point (pH 6.2) (Hu and McClements, 2015).

In the case of ionic strength and pH, these nanoparticles have been shown to be highly liable to aggregation at a low concentration of sodium chloride (NaCl), and to be unstable at pH above 5. The salt added to formulations increases the ionic strength, with consequent increases in van der Waals interactions and hydrophobic effects among the protein chains, favoring aggregation and precipitation of the proteins (Dai et al., 2016). Similar findings have been reported in other studies (Patel et al., 2010).

Given that the technology of nanoencapsulation in zein particles offers considerable potential for the development of formulations capable of improving the properties of the encapsulated compounds, it is essential to develop strategies to improve the chemical stability and extend the shelf life of these systems. Aspects to consider include the particle size, polydispersity index, encapsulation efficiency, and release of the active agent over an extended period. These issues are often not discussed or presented in the published studies, but they are vital for the development of commercial products based on zein nanoparticles.

## Strategies to improve the stability of zein nanoparticles

Several strategies have been reported for improving the stability of zein nanoparticles (Table 1). However, it should be stressed that there have been few relevant published studies concerning this issue.

**Table 1:** Studies found in the literature concerning strategies to improve the stability of zein nanoparticles, and the results obtained.

**Zein NPs (ZNPs)**	**Strategies to improve stability**	**Stability results**	**References**
5-fluorouracil ZNPs	Formulation stored at 4°C	ZNPs aggregated after 6 months at 25°C, but not at 4°C.	Lai and Guo, 2011
Thymol ZNPs	Coated with caseinate and chitosan	Coating the ZNPs increased the encapsulation efficiency. At low concentrations of emulsifiers, ZNPs agregated.^*^	Zhang et al., 2014
Mint oil ZNPs	Coated with gum arabic	Coated ZNPs were stable at pH 3 to 8; uncoated ZNPs released the oil faster.^*^	Chen and Zhong, 2015
Thymol and carvacrol ZNPs	Formulation stored at 4°C	ZNPs precipitated after 2 months at 20°C, but not at 4°C.	da Rosa et al., 2015
Resveratrol ZNPs	Coated with sodium caseinate	Coating the ZNPs improved their stability, considering the effects of ionic strength, pH, and temperature during storage for 28 days.	Joye et al., 2015
Lutein ZNPs	Coated with lecithin and Pluronic	Coating the ZNPs improved their chemical stability during 30 days, compared to uncoated ZNPs.	Chuacharoen and Sabliov, 2016
Hollow ZNPs	Thermal treatment in a thermostatic water bath	ZNPs with treatment at 75°C for 15 min presented a smaller mean diameter and lower polydispersity index.^*^	Sun et al., 2016
Hollow ZNPs	Coated with carrageenan	Coated ZNPs maintained a constant average diameter during storage at pH between 5.25 and 6.75 for 30 days; uncoated ZNPs precipitated.	Cheng and Jones, 2017
Resveratrol ZNPs	Coated with pectin	The stability was influenced by the pectin concentration.^*^	Huang et al., 2017

(*) *No evaluation was made of the temporal stability of these particles*.

### Storage conditions

Studies of zein nanoparticles have found that their stability varies according to the way that the formulation is stored. Gomez-Estaca et al. (2012) reported that a formulation of zein nanoparticles with curcumin remained stable for 3 months when stored in the dark. The stability was not evaluated over an extended period or in the presence of light, which would be very important due to the photosensitivity of the active compound. Lai and Guo (2011) and da Rosa et al. (2015) produced zein nanoparticles containing active agents such as 5-fluoracil, thymol, and carvacrol and found that formulations kept at 20°C presented precipitation and aggregation after 2 and 6 months of storage. However, nanoparticles kept at 6°C remained stable over the same periods of time. No explanations were provided for the differences in behavior according to temperature.

### Thermal treatment

Sun et al. (2016) subjected zein to thermal treatment in a thermostatic water bath before synthesis of the nanoparticles, using different conditions of time and temperature, in order to modify the characteristics of the material and increase its denaturation temperature. Zein particles produced with treatment at 75°C for 15 min presented a smaller mean diameter and lower polydispersity index. However, when longer periods of time and higher temperatures were employed, the mean diameter and polydispersity index of the nanoparticles increased. No evaluation was made of the stability of the formulations as a function of time. Selling et al. (2007) investigated the effects of temperatures in the range 25–70°C on the secondary and tertiary structures of zein. It was found that treatment at 70°C for 15 min caused changes in the primary structures and decreased the alpha-helix content of the secondary structure. These alterations were reversed when the temperature returned to 25°C. In contrast, Cabra et al. (2006) reported irreversible changes in the alpha-helix structures of zein proteins after treatment at 90°C.

These results suggest that a short heat treatment (15 min) partially unravels the tertiary structures of zein molecules, resulting in a monodisperse formulation with smaller nanoparticle size. Heat treatment for longer times and at higher temperatures leads to complete unraveling of the zein molecules, which can then aggregate, hence increasing interactions among the polypeptide chains of different protein molecules (Sun et al., 2016). However, there are limited studies about the heat-induced structural and physicochemical changes of alcohol-soluble proteins.

### Coatings

Solutions proposed for overcoming the problems of aggregation and precipitation of zein nanoparticles have involved coating the particles with emulsifiers such as carrageenan, gum arabic, lecithin, Pluronic, sodium caseinate, pectin, and chitosan, in order to maintain repulsion among the particles (Luo et al., 2011; Chuacharoen and Sabliov, 2016; Cheng and Jones, 2017; Huang et al., 2017). As an example, the use of carrageenan was found to maintain a constant average diameter of zein nanoparticles stored at pH between 5.25 and 6.75 during 30 days, while uncoated nanoparticles showed significant precipitation (Cheng and Jones, 2017). Coating with gum arabic resulted in stability of zein nanoparticles loaded with mint oil in an extended pH range from 3 to 8, while release of the oil was faster at lower pH (Chen and Zhong, 2015). However, no evaluation of the temporal stability of these particles was made. Chuacharoen and Sabliov (2016) coated nanoparticles composed of zein and lutein with lecithin and Pluronic. The coated particles presented a larger average diameter, compared to particles not coated with the emulsifiers, together with improved performance in the chemical stability parameters evaluated during 30 days in the presence of ultraviolet light. Joye et al. (2015) reported that the coating of zein nanoparticles with sodium caseinate was effective in improving the stability of nanoparticles loaded with resveratrol, considering the effects of ionic strength, pH, and temperature during storage for 4 weeks. In a study of synthesized zein nanoparticles loaded with resveratrol and coated with pectin, Huang et al. (2017) reported an important influence of the pectin concentration on the stability of the formulation, although no data were provided for the chemical stability of the particles according to time, temperature, or pH. The average diameter and zeta potential of the nanoparticles were shown to be dependent on the pectin concentration, with the nanoparticles aggregating and forming precipitates at lower pectin concentrations. This effect of emulsifier concentration on the chemical stability was reported previously by Zhang et al. (2014), who used sodium caseinate and chitosan to increase the encapsulation efficiency and improve the antimicrobial activity of thymol contained in the formulation. The substantial aggregation and sedimentation of the nanoparticles at low concentrations of emulsifiers could be due to the low electrical potentials on the particles, resulting in weak electrostatic repulsion among them. It is also possible that an emulsifier molecule could bind to two or more zein nanoparticles, forming bridges and causing precipitation of the formulation (Hu and McClements, 2015).

### Protein and emulsifier concentrations

Zhang et al. (2014) and Huang et al. (2017) described directly proportional relations between the zein concentrations used to produce the particles and their mean diameters, while inversely proportional relations were obtained between the emulsifier concentrations and the mean particle diameters, with the smallest nanoparticles being produced using a combination of Pluronic and lecithin.

### Ionic strength and pH

The results obtained for the influence of ionic strength disagreed with the findings of Joye et al. (2015), since there was no evidence of any effect of ionic strength on the synthesis of the particles. When the pH of the aqueous phase was increased from 2 to 7.4, the mean diameter and polydispersity index values decreased, while when the pH was further increased from 7.4 to 10, the diameter and polydispersity of the particles increased. However, no information was provided concerning the final pH values or the temporal stabilities of the formulations.

Considering the findings of the published studies, we had summarized how the factors affects the colloidal stability of zein nanoparticles (Supplementary Figure 1). Also, Supplementary Figure 2 (see supplementary material) recommended a strategy for improving the stability of zein nanoparticles such as thermal treatment as well as the use of coatings employing other biopolymers such as pectin, chitosan, caseinate, and others, or lipids such as lecithin. However, each case should be analyzed individually, since if one of the objectives of preparing the nanoparticles is to produce a carrier system for bioactive compounds, it is necessary to consider possible interactions of the active agent with the components of the coating.

## Concluding remarks

This article presents and describes the methods used to produce zein nanoparticles, as well as the main issues concerning the colloidal stability of these particles and ways to improve their stability. It was found that there have been few detailed studies of the temporal stability of these particles present in solution. It is notable that a considerable number of studies have reported low stabilities of the formulations, which limits the production of zein nanoparticles on a large scale for commercial purposes. Several techniques have been employed to overcome this difficulty, in order to benefit from the advantages offered by encapsulation employing zein nanoparticles.

Therefore, it is strongly recommended that a detailed study should be undertaken for each type of particle that it is intended to prepare. Experimental design tools can be employed to optimize preparation conditions in order to produce nanoparticulate systems with good colloidal characteristics. A number of important factors always must be considered during this optimization, in order to maximize the temporal stability of the system. These include polymer and emulsifier concentrations, pH, ionic strength, thermal treatment, coatings, temperature, and the presence of illumination during storage. Furthermore, it is essential to evaluate and publish the colloidal stability over extended periods. Such procedures are of great importance for the feasibility and reproducibility of processes used to produce zein nanoparticles with the aim of developing scalable processes for their commercial manufacture. The marketing of products with shelf lives that are in accordance with commercial requirements can bring benefits for various purposes, justifying the investment in research carried out on this subject.

## Author contributions

MP, RdL, and LF proposed and wrote the manuscript.

### Conflict of interest statement

The authors declare that the research was conducted in the absence of any commercial or financial relationships that could be construed as a potential conflict of interest.
