# Supplementary material for: Zein Nanoparticles and Strategies to Improve Colloidal Stability: A Mini-Review
Source: Front Chem. 2018 Jan 25;6:6. doi: 10.3389/fchem.2018.00006 (PMC5810256; doi:10.3389/fchem.2018.00006)
Supplement: Supplementary file 1 [file Table1.docx]

Supplementary Material

**Zein nanoparticles and strategies to improve colloidal stability: a mini-review**

Mônica Pascoli^1^, Renata de Lima^2^, Leonardo Fernandes Fraceto^1*^

^1^São Paulo State University (UNESP), Institute of Science and Technology, Sorocaba, SP, Brazil

^2^Department of Biotechnology, University of Sorocaba, Sorocaba, SP, Brazil

* Correspondence: Corresponding author: [leonardo@sorocaba.unesp.br](mailto:leonardo@sorocaba.unesp.br)


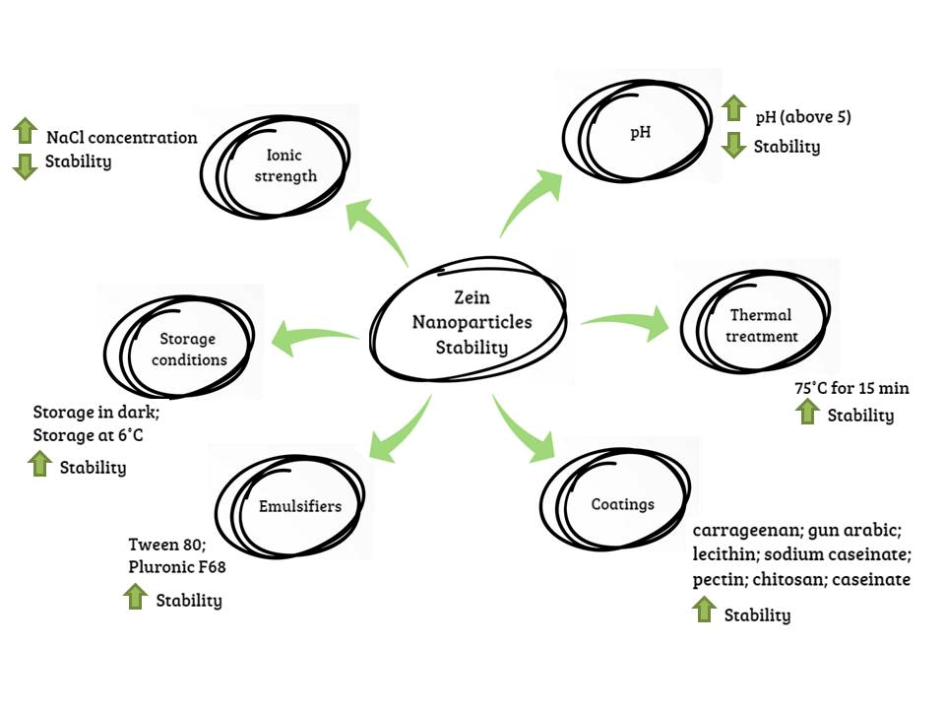


Supplementary Figure 1: Summary of parameters that influence the zein nanoparticle stability: i) an increase in ionic strength decreases the nanoparticle stability; ii) an increase in pH above of 5 decrease the nanoparticles stability; iii) the use of thermal treatment increase the nanoparticle stability (75^o^C for 15 min); iv and v) coating agents and emulsifiers increase the nanoparticle stability in solution; vi) storage conditions in dark and at 6^o^C increase the colloidal nanoparticle stability.


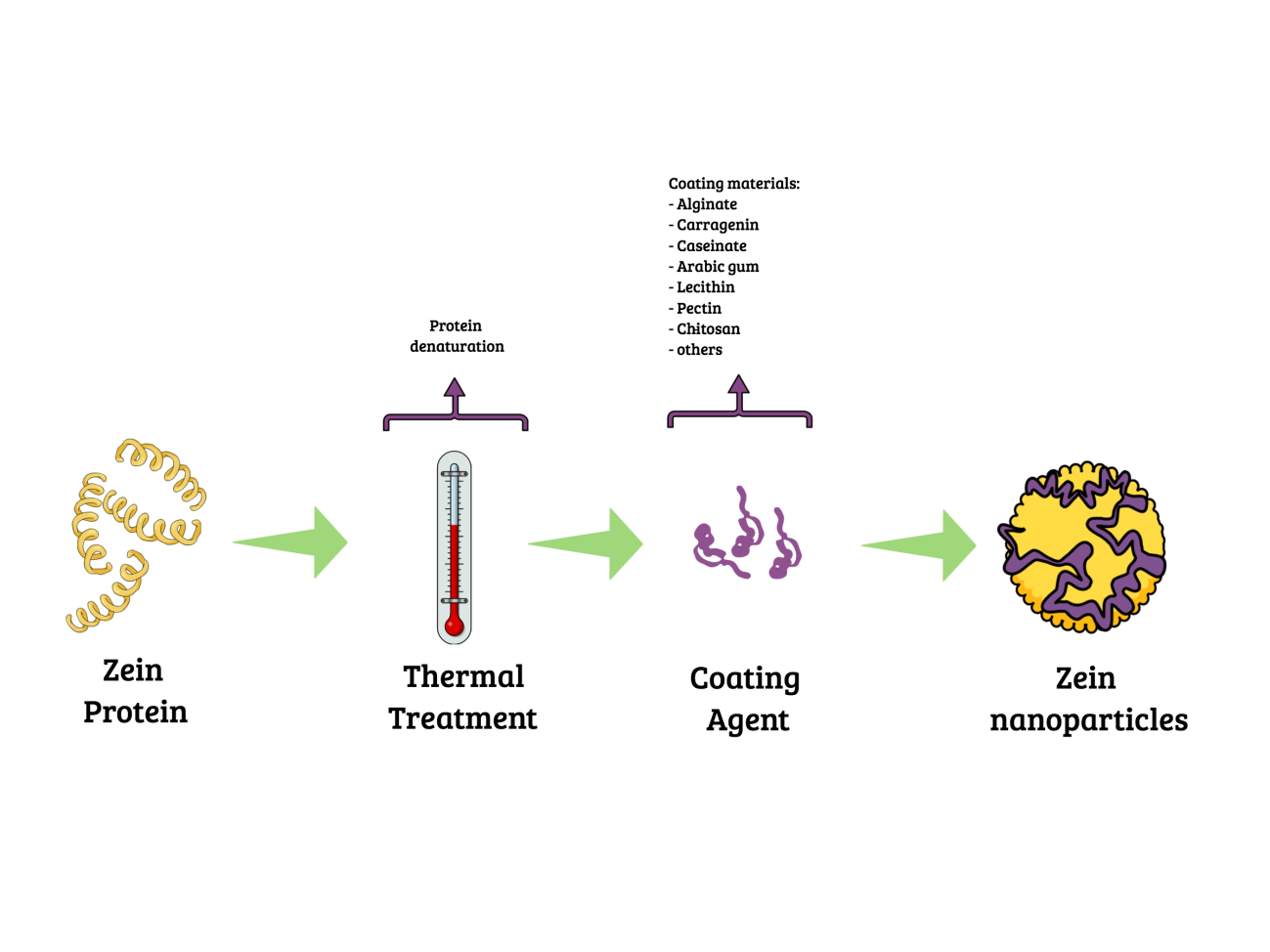


**Supplementary Figure 2:** Proposed strategies to be used to increase the stability of zein nanoparticles. At the first moment is need to do the thermal treatment (protein denaturation) and after the use of coating agent in order to improve the zein nanoparticle stability.

Supplementary Table 1:Studies found in the literaturerelated to the production of zein nanoparticles as carriers for different active agents. The results and preparation methods employed are summarized. Note the absence of information concerning the stabilities of some of the formulations.

| Zein NPs (ZNPs) | **Size (nm)** | **Encapsulation**  **efficiency (%)** | **Method of**  **preparation** | **Stability** | **References** |
| --- | --- | --- | --- | --- | --- |
| Curcumin ZNPs Curcumin zein-shellac NPs | 118 - 240.2 62.9 - 4280 | 82.7 93.2 | Antisolvent precipitation | - | Sun et al.(2017) |
| Curcumin ZNPs | 175 - 900 | 85 - 90 | Electrohydrodynamic atomization | 3 months in dark conditions | Gomez-Estacaet al.(2012) |
| Cranberry procyanidin ZNPs | 392-447 | 48 - 79 | Liquid-liquid dispersion | - | Zou et al. (2012) |
| Thymol and carvacrol ZNPs | 51.9 - 328.1 | > 50 | Liquid-liquid dispersion | - | Wu et al. (2012) |
| 5-fluorouracil ZNPs | 114.9 | 60 | Phase separation | ZNPs aggregated after 6 months at 25°C, but not at 4°C | Lai andGuo(2011) |
| Catalase and superoxide dismutase ZNPs | 255.21 | 31.36 - 44.99 | Phase separation | - | Lee et al. (2013) |
| Thymol and carvacrol ZNPs | 108 - 122 | 88.5 - 99.9 | Antisolvent precipitation | ZNPs precipitated after 2 months at 20°C, but not at 4°C | Da Rosa et al.(2015) |
| Chitosan coated retinol ZNPs | Retinol ZNP: 300 Chitosan retinol ZNP: 500 | 64.9 80 | Antisolvent precipitation | - | Park et al. (2015) |

**REFERENCES**

da Rosa, C. G., de Oliveira Brisola Maciel, M. V., de Carvalho, S. M., de Melo, A. P. Z., Jummes, B., da Silva, T., et al. (2015). Characterization and evaluation of physicochemical and antimicrobial properties of zein nanoparticles loaded with phenolics monoterpenes. *Colloids Surf. Physicochem. Eng. Asp.* 481, 337–344. doi:10.1016/j.colsurfa.2015.05.019.

Gomez-Estaca, J., Balaguer, M. P., Gavara, R., and Hernandez-Munoz, P. (2012). Formation of zein nanoparticles by electrohydrodynamic atomization: Effect of the main processing variables and

suitability for encapsulating the food coloring and active ingredient curcumin. *Food Hydrocoll.* 28, 82–91. doi:10.1016/j.foodhyd.2011.11.013.

Lai, L. F., and Guo, H. X. (2011). Preparation of new 5-fluorouracil-loaded zein nanoparticles for liver targeting. *Int. J. Pharm.* 404, 317–323. doi:10.1016/j.ijpharm.2010.11.025.

Lee, S., Alwahab, N. S. A., and Moazzam, Z. M. (2013). Zein-based oral drug delivery system targeting activated macrophages. *Int. J. Pharm.* 454, 388–393. doi:10.1016/j.ijpharm.2013.07.026.

Park, C.-E., Park, D.-J., and Kim, B.-K. (2015). Effects of a chitosan coating on properties of retinol-encapsulated zein nanoparticles. *Food Sci. Biotechnol.* 24, 1725–1733. doi:10.1007/s10068-015-0224-7.

Sun, C., Xu, C., Mao, L., Wang, D., Yang, J., Gao, Y. (2017). Preparation, characterization and stability of curcumin-loaded zein-shellac composite colloidal particles. *Food Chem*. 228, 656-667. doi: 10.1016/j.foodchem.2017.02.001.

Wu, Y., Luo, Y., and Wang, Q. (2012). Antioxidant and antimicrobial properties of essential oils encapsulated in zein nanoparticles prepared by liquid–liquid dispersion method. *LWT - Food Sci. Technol.* 48, 283–290. doi:10.1016/j.lwt.2012.03.027.

Zou, T., Li, Z., Percival, S. S., Bonard, S., and Gu, L. (2012). Fabrication, characterization, and cytotoxicity evaluation of cranberry procyanidins-zein nanoparticles. *FoodHydrocoll.* 27, 293–300. doi:10.1016/j.foodhyd.2011.10.002.
